# A path forward for the implementation of shared decision-making in valvular heart disease: global joint recommendations from clinicians, patients and researchers

**DOI:** 10.1186/s12961-025-01393-x

**Published:** 2025-10-16

**Authors:** Sandra B. Lauck, Martha Gulati, Krystina B. Lewis, Nicola Straiton, Johanna J. M. Takkenberg, Peyman Sardari Nia, Sandra McGonigle, Karen Padilla, Ellen Ross, Hélène Eltchaninoff, Bernard Prendergast

**Affiliations:** 1 University of British Columbia, T201-2211 Wesbrook Mall, Vancouver, BC V6T 2B5 Canada; 2 Cedars-Sinai Medical Center’s Smidt Heart Institute, Los Angeles, California United States of America; 3 School of Nursing, Faculty of Health Sciences, University of Ottawa; University of Ottawa Heart Institute; Centre for Implementation Research, Ottawa Hospital Research Institute, Vancouver, Canada; 4 Nursing Research Institute, St Vincent’s Health Network Sydney, St Vincent’s Hospital Melbourne, Australian Catholic University, New South Wales, Australia; 5 School of Nursing, Midwifery and Paramedicine, Australian Catholic University, Sydney, Australia; 6 Department of Cardio-Thoracic Surgery, Erasmus University Medical Center, Rotterdam, The Netherlands; 7 Department of Cardiothoracic Surgery, Maastricht University Medical Center, Maastricht, The Netherlands; 8 London, United Kingdom; 9 Global Heart Hub, Dublin, Ireland; 10 Heart Valve Voice, Ottawa, Canada; 11 Department of Cardiology, University Hospital of Rouen, Rouen, France; 12 Thomas’ Hospital and Cleveland Clinic, London, United Kingdom

**Keywords:** Shared decision-making, Valvular heart disease, Health policy, Knowledge translation

## Abstract

**Background:**

Shared decision-making (SDM) is widely endorsed in international guidelines for the treatment of valvular heart disease (VHD). Despite evidence that the process improves outcomes and does not increase the burden of consultations, SDM has not been adopted as a standard of care across regions and diverse health systems.

**Methods:**

We conducted a 3-phase study co-led by clinicians and people with lived experience using an integrated knowledge translation approach guided by the knowledge-to-action framework. In a preparatory phase, we conducted exploratory semi-structured interviews with 19 international and diverse experts to identify barriers and enablers to SDM in VHD; we used thematic analysis to identify the major issues to inform project development. We convened an in-person meeting of patients and patient advocates (*n* = 9), clinicians (*n* = 11) and researchers (*n* = 3) from 10 countries to build joint recommendations. Lastly, we conducted a series of local and international meetings to validate the findings and inform future initiatives.

**Results:**

Challenges identified included (1) concerns about clinicians’ availability and time requirements, (2) uncertainty about how to practice SDM and (3) absence of regional data to evaluate SDM in VHD. The joint recommendations clustered on five global areas of focus and six sets of recommendations tailored to regional contexts and cultural norms. Final recommendations on (1) preparing patients and carers, (2) training healthcare teams and (3) creating a supportive system were further enhanced by VHD knowledge users’ input in various regional settings.

**Conclusions:**

This first report co-led by diverse stakeholders offers a practice and policy-ready roadmap to strengthen the implementation and evaluation of SDM in VHD.

## Introduction

Patients facing a decision about the treatment of their valvular heart disease experience a complex journey of care from diagnosis to one or more treatments, and a lifetime of management. From the perspective of patients and people who support them, this pathway can be uncertain, challenging and inequitable [1, 2]. International advocacy and clinical and policy efforts are under way to improve the detection, diagnosis and timely treatment of valvular heart disease to address these barriers to access to timely treatment [3, 4]. In parallel, efforts are required to change the culture of care to strengthen the partnership between patients and their healthcare providers to achieve high-quality treatment decisions [5–7]. Recent guidelines have widely endorsed shared decision-making as an essential component to achieve optimal outcomes in the rapidly evolving domain of clinical care of valvular heart disease [8, 9]. However, implementing agreements and processes in clinical practice and health policy remains difficult for multiple reasons, preventing this approach from becoming a standard of care across regions.

Shared decision-making (SDM) is the relatively simple concept of a bi-directional and collaborative process of information exchange between patients and clinicians to reach a considered and beneficial decision about treatment options informed by, and based on individuals’ values and preference, including the choice to not undergo treatment [10]. Importantly, an SDM conversation goes beyond the conventions of patient education, which often focuses on providing specific information concerning one treatment option and satisfies the requirement of informed consent – the transactional and contractual agreement that enables clinicians to carry out the recommended treatment [11]. When clinicians practice SDM, they intentionally invite patients to communicate their goals of care, including their priorities, values and preferences. During these interactions, clinicians make patients aware that they have a choice and present treatment options and discuss their potential benefits and risks tailored to the individual patient. In this way, the aim is to reach a shared treatment decision – one that incorporates clinicians’ knowledge of the condition and best available evidence, prognosis, treatment options and possible outcomes in the unique clinical context of the patient and acknowledges the patient’s expertise in knowing what matters most to them, the impact of their health condition on their daily life and expectations and preferences for the possible outcomes [12, 13].

For patients and their families, the meaningful and effective use of SDM may contribute to improved outcomes. There is growing evidence that SDM interventions, including the use of patient decision aids developed according to international standards [14, 15], reduce decisional conflict, increase patient knowledge and satisfaction with care and enhance engagement in their health [16]. When patients perceive that SDM has been achieved, they are more knowledgeable and satisfied with their care [17]. For clinicians and policy-makers, SDM supports the shift from clinician-driven and procedure-based care, and allows movement beyond the deficit and disease model of health to a more person-centred and lifetime disease/health-focused culture of care [18].

Despite guideline endorsement, there remains a gap between the good intentions of SDM and progress in changing the conversation and the delivery of care to achieve these goals. The question of how to do SDM within health systems remains unanswered among clinicians and across international regions. This is particularly salient in the rapidly evolving clinical context of the treatment of valvular heart disease, where there is a lag between guideline-driven care and emergence of new treatment options and associated intense research activities. To address this challenge, we must prioritize efforts to develop, implement and evaluate strategies to support the effective implementation of SDM into practice, and remove the significant barriers to making cultural changes in the delivery of valvular heart disease care.

### From good intentions to implementation: a call to action for clinicians and patients

The involvement of people with lived experiences is crucial to developing pathways towards SDM implementation. This builds on evidence that patients want to actively participate and choose appropriate treatments that are right for them [19], and co-lead research, policy and clinical collaborations that yield sustained change in practice and knowledge exchange [20]. In addition, local and international patient advocacy organizations play an increasing role in developing collaborative networks that leverage the close partnerships of patients, caregivers and families, and clinicians, researchers and policy-makers to collectively inform clinical care, drive research agendas and promote equitable and person-centred health policy [21, 22].

The Global Heart Hub (GHH) is an alliance of cardiac patient organizations that aims to create a unified voice for people with (or affected by) heart disease to advocate for the best possible local, national and international outcomes. Prompted by issues raised by global organizations representing people with valvular heart disease, GHH prioritizes the empowerment of patients to engage in decision-making about their care. This resulted in the publication of two unique consensus documents that promote collective efforts to improve the patient care journey globally [23] and in Europe [24], provide a guide to patient engagement in SDM, and equip patients with contemporary knowledge and effective tools developed in collaboration with clinicians [25]. Although these consultations raised awareness and levels of engagement, these initiatives uncovered evidence that a unidirectional distribution of education resources was insufficient to strengthen the global implementation of SDM. In response, GHH built on the success of this initiative to accelerate efforts to develop strategies and promote local and global actions aimed at the meaningful adoption of SDM across the international continuum of valvular heart disease care and prioritized the leadership of people with lived experience and diverse stakeholders. We report on the consensus findings and processes employed to co-develop a global roadmap to the successful implementation of SDM in valvular heart disease that will support the adoption of international guidelines.

## Methods

### Integrated knowledge translation

We used an integrated knowledge translation (iKT) approach to guide the work. iKT is an increasingly adopted research approach that aims to engage knowledge users as equal partners in the co-production of new knowledge using engagement and co-production of process design, development and dissemination of findings and outreach [26, 27]. We were further guided by the Knowledge-to-Action (KTA) framework that provides conceptual clarity about the key elements of moving evidence into practice and leverages existing and emerging knowledge to inform actions and activities to adapt to local contexts, identify and address barriers and facilitators to change and drive evaluation [28].

We established the co-leadership of the initiative, including a patient with lived and living experience of disease management and treatment decisions and a strong track record of advocacy in partnership with GHH (S.McG.) and a clinician with expertise in patient-centred clinical program development and care of people with valvular heart disease (S.B.L.). Together, we identified a diverse and representative group of international patients, researchers, policy-makers and clinicians, including physicians and nurses. At the onset, we recognized the inherent challenges of facilitating a truly global effort with representation from low-, middle- and high-income countries and health systems that differ significantly in resources, priorities and access to care. Although acquired and congenital valvular heart disease and their associated risk factors represent a global health issue, intersectional factors, such as racism, sexism, classism and ageism [29, 30], further augment the impact of socioeconomic determinants of health and illustrate systems of power that drive inequities in the diagnosis and management of valvular heart disease. With this limitation and context in mind, we formed a core group inclusive of knowledge users and producers who supported all aspects of the initiative in partnership with GHH and provided consent to the dissemination of the project for knowledge translation purposes.

### Phase 1: Preparatory work

In keeping with the guidance of the KTA framework, we conducted a comprehensive review of contemporary literature concerning the pivotal role of SDM in the management of valvular heart disease, barriers to effective implementation and potential strategies to promote change in clinical care and health policy. In addition, we conducted in-depth semi-structured exploratory interviews with 19 experts, representing people with valvular heart disease, cardiologists, cardiac surgeons, nurses and researchers to establish our collaboration and gain preliminary perspectives on personal and professional experiences of the challenges, facilitators and opportunities to accelerate the implementation of SDM (Table 1). We used an exploratory thematic analysis to identify key preliminary findings [31]. We subsequently produced a discussion paper to disseminate the findings, establish a collective common understanding of current state of knowledge, adoption and barriers and provide preliminary scaffolding for the development of consensus recommendations. The report was distributed as a structured pre-reading discussion paper in advance of the policy roundtable to all participants. In addition, the delegate preparation package included an overview of the meeting’s objectives and a preliminary introduction to the diverse participants to convey the central importance of patient input and perspectives, and the anticipation of diverse disciplinary representation at the meeting to help clarify expectations.

**Table 1:** Framework used to gain preliminary perspectives and inform development of meeting agenda for phase 2

Probe: From your perspective, what is shared decision-making and why does it matter?
Probe: What are the unique challenges of shared decision-making in valvular heart disease?
Guidance to prompt discussion based on literature: - Capacity and time - Education, guidance and resources - Evidence and evaluation
Probe: What global and local activities are necessary to advance the implementation of shared decision-making in valvular heart disease?
Guidance to prompt discussion based on literature: - Role of advocacy, education and support - Training of healthcare professionals - Integration of discussions in journey of care - Role of health policy - Role of research and knowledge translation
Probe: What would success look like at the planned priority-setting meeting?

### Phase 2: Joint recommendation policy roundtable

**Fig. 1:**
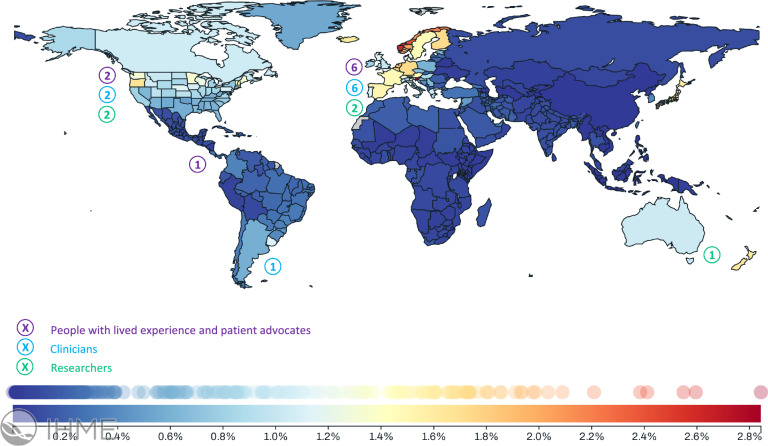
Country of origin and role of participants in the context of global burden of nonrheumatic valvular heart disease (both sexes, all ages, 2021, percentage of total deaths). Source: Global Burden of Disease study, Institute for Health Metrics and Evaluation (University of Washington, Seattle, United States) (https://vizhub.healthdata.org/gbd-compare/#) in collaboration with *The Lancet* (https://www.thelancet.com/gbd/about)

In August 2023, we convened an in-person meeting of 23 international experts, representing patients, clinicians and researchers from 10 countries (Europe, 14; North America, 6; Central and South America, 2; Australia, 1), including 9 people with lived and living experience and/or patient advocates, 9 clinicians (referring cardiologists, 2; interventional cardiologists, 4; cardiac surgeons, 2; nurses, 1) and 5 researchers. Figure 1 shows the country of origin of all participants and their role in the project, projected against available data on the mortality associated with nonrheumatic valvular heart disease generated by the Global Burden of Disease (DBD) Study conducted at the Institute for Health Metrics and Evaluation (IHME, University of Washington, Seattle, United States) [32]. In this context, we recognise that (1) the global impact of valvular heart disease remains unevenly documented and reported across global representation and (2) that there is a disproportionate representation of participants from high-income countries in our project.

The agenda was co-designed by the core group with the objectives of (1) reviewing the findings of phase 1 and (2) building joint recommendations. We structured the dialogue and consensus building in a series of facilitated break-out sessions and group discussions designed to build on the findings that emerged from the preparatory interviews, prioritize the inclusion of diverse perspectives and achieve actionable recommendations. We aimed to harness global insights and develop an action plan relevant to international regions and potentially tailored to diverse health systems and cultural contexts. The meeting was facilitated by the co-chairs in partnership with GHH with the support of Health Policy Partnership who led the preparatory and coordinating activities.

### Phase 3: Validation of recommendations

We engaged with patient organizations across international regions to further ascertain diverse perspectives and plan knowledge translation activities. We were invited to present the early consensus recommendations on the development of the roadmap at the PCR London Valves scientific meeting. Discussion centred on navigating challenges in SDM for clinicians and patients. In this setting, we aimed to validate our discussions and gain further insights on strengthening the proposed road map to implementation. We integrated key perspectives in the final global recommendations.

## Results

### Perspectives on the challenges in the unique context of valvular heart disease

The completion of phase 1 resulted in a thematic analysis of the preliminary key informant interviews. The data yielded three preliminary themes that described various perspectives on the challenges concerning SDM in valvular heart disease: (1) patients’ perception that clinicians lack time to engage in SDM, and clinicians’ concerns about their capacity for SDM whilst managing the competing demands of their significant clinical workload; (2) patients’ experiences of barriers to accessing high-quality, timely and tailored information about valvular heart disease, and clinicians’ uncertainty about “how to do SDM” in an effective way; and (3) the absence of regional data and global evidence to evaluate the implementation, effectiveness and efficiency of SDM in clinical settings to meet the unique and evolving context of valvular heart disease.

### Development of joint recommendations

During phase 2, the following pillars guided the group’s roundtable and small group discussions: (1) patient information, resources and education, support and active involvement, (2) training and support for clinicians and (3) embedding SDM in national care pathways and government policy. Stakeholders unanimously noted the need for both local and international action to accelerate implementation activities, leverage the collective momentum and sustain success. The group reached consensus on five key areas for united global action, and six local calls to action.

Global areas of focus: Multiple regions experience similar challenges to the implementation of SDM. Global action is a strategy to leverage international experiences and accelerate momentum on the following issues:

1. Develop a common understanding of the components required for quality SDM: SDM requires an invitation for people receiving care to be actively included and engaged in an informed conversation and a collaborative process to reach a joint decision with clinicians. Importantly, there is global consensus that SDM goes beyond patient education, information sharing and the confirmation of consent: the process must acknowledge patients’ expertise concerning the impact of their health condition on their daily life, their values and their preferred outcomes.
2. Increase the availability of data and research on the effectiveness of SDM and implementation strategies: Globally, there is limited evidence reporting the specific impact of SDM specifically in valvular heart disease. Furthermore, there is a pressing need to drive an international agenda to study the barriers and facilitators of implementation across regions to select tailored implementation strategies that will support effective and sustained change in the culture and processes of care.
3. Strengthen a multidisciplinary care approach: Equitable access to a comprehensive Heart Team is a central tenet of the management of valvular heart disease and is ideally positioned to support the journey of care so that patients can consider all treatment options.
4. Promote the incorporation of SDM in health policies: SDM should be viewed as a fundamental right of people receiving care. In the context of valvular heart disease, guidelines are insufficient to ensure that all people have access to this level of inclusion in their treatment decisions. Advocacy efforts are needed to engage policy-makers and explore strategies such as funding models, policy priorities and legislation.
5. Develop strong partnerships with key stakeholders: The change required to make a sustained difference for people living with valvular heart disease depends on global collective efforts and strong collaborations that include patient and carer representatives, heart patient support organizations, clinicians, researchers with expertise in implementation science, health system leaders and hospital administrators. Strengthening this network of partnerships is essential to support the implementation of SDM across regions and within systems.

Local areas of focus: Recognizing the importance of tailored strategies to address cultural norms, regional contexts and other imperatives, local areas of focus can help overcome diverse barriers and leverage health system assets to strengthen the adoption of SDM.

1. Develop comprehensive and accessible local-level resources to support the trajectories of care for people with valvular heart disease: Regionally-adapted information that provides people with information that equips them to better understand the journey of care for valvular heart disease is lacking. Topics particularly salient to local needs include clarified expectations of disease progression and typical care pathways, preparing for a treatment decision, support requirements, recovery and rehabilitation, and lifetime management.
2. Increase the availability and implement and evaluate the use of culturally-appropriate and high-quality patient decision aids: Patients and clinicians require evidence-based tools that are designed according to international standards to inform SDM interactions. When designed appropriately [33], patient decisions aids are effective resources that combine information on treatment options and potential outcomes, benefits and risks that aim to support people to think about what matters most to them when deciding on their preferred treatment (including whether to have treatment at all). These tools must meet the diverse needs of people with lived and living experience, tailored to diverse levels of health literacy and cultural context and available in diverse formats, including paper and digital resources [16]. To address current gaps in diverse regions, there is a pressing need to prioritize the development and implementation of evaluation of patient decision aids to improve the treatment of valvular heart disease.
3. Improve training and competencies in SDM for healthcare professionals: Across regions, clinicians report their agreement with the intent of SDM but highlight limited competencies and lack of exposure to quality learning opportunities. Locally, the provision of clinical education across the continuum of learning (from initial training to continuous education) and increased exposure at scientific meetings offer an opportunity to strengthen clinicians’ capacity to engage effectively in SDM. To achieve this goal, clinicians must be supported to evaluate the quality and impact of SDM in a meaningful way within their services.
4. Seek to include distinct steps within local care pathways where individuals’ preferences, values and priorities are understood and captured: The objectives of SDM interface with multiple aspects of healthcare delivery that require supportive systems. To this end, local efforts are required to encourage people to discuss their preferences, values and priorities. The availability of multiple opportunities and time points for people to engage with clinicians (including primary care, treating physicians and nursing professionals) will strengthen a system supportive of SDM. Care pathways need to be designed in ways that provide options for patients to consider all options, including the choice to opt for no treatment if efforts may be futile or incompatible with their priorities and goals of care.
5. Encourage investment in research to identify and scale-up best practices for SDM in valvular heart disease: Local efforts are needed to prioritize, fund and disseminate research that validates SDM tools and models of care and improves understanding of evidence-informed implementation strategies to support sustained SDM in practice.
6. Advocate for the adoption of novel funding or reimbursement models in healthcare to help shift the culture of care from procedure-driven to a more person-centred and disease-focused approach: Historical policy and funding models continue to entrench clinicians in the provision of siloed care, including patients’ experience of their first encounter with a specialist (e.g. multidisciplinary clinic versus surgical services versus interventional cardiology). Health systems that ultimately incentivize volumes of procedures rather than the quality of patients’ experiences may be another pertinent example of this systemic barrier to prioritizing SDM. Exploring novel funding models that drive change in processes and incorporate SDM may strengthen the inclusion of patients’ perspectives.

**Fig. 2:**
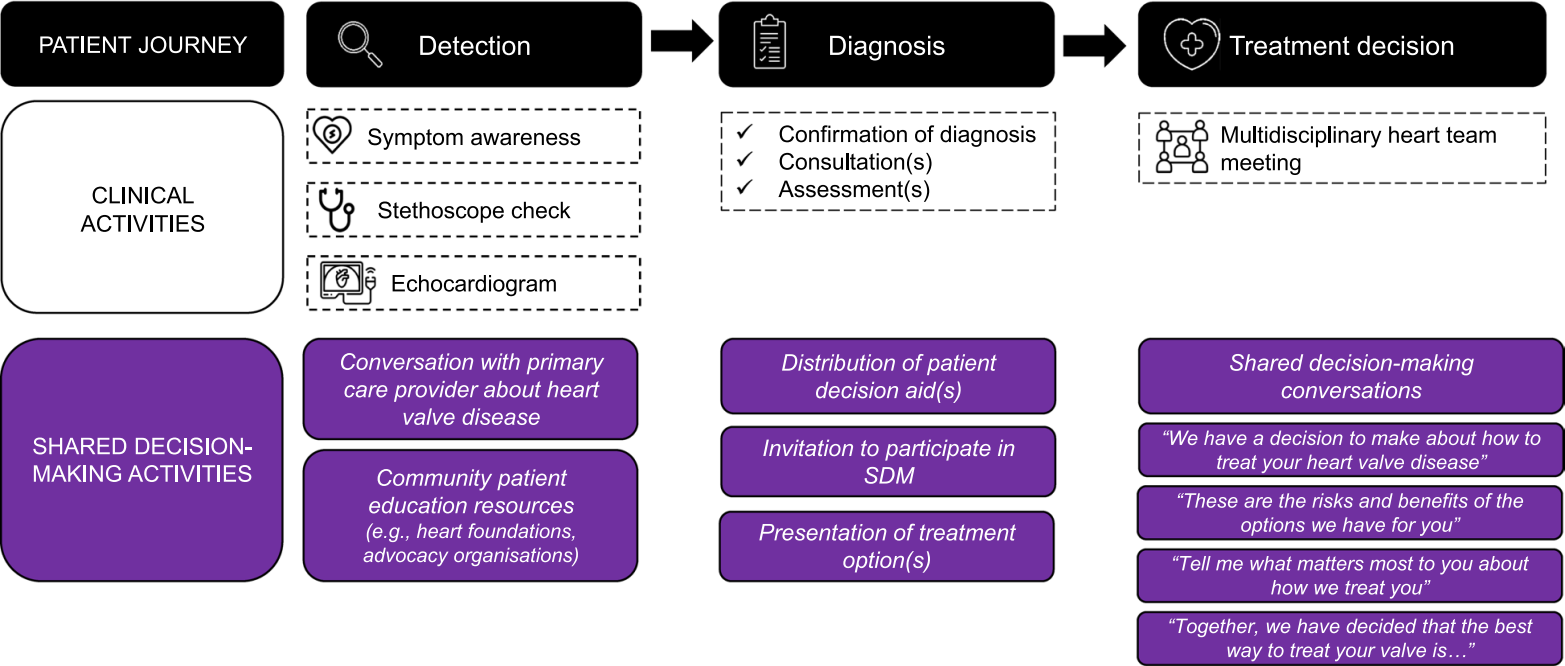
Opportunities for integration of shared decision-making activities from detection to treatment in valvular heart disease patient pathway

**Fig. 3:**
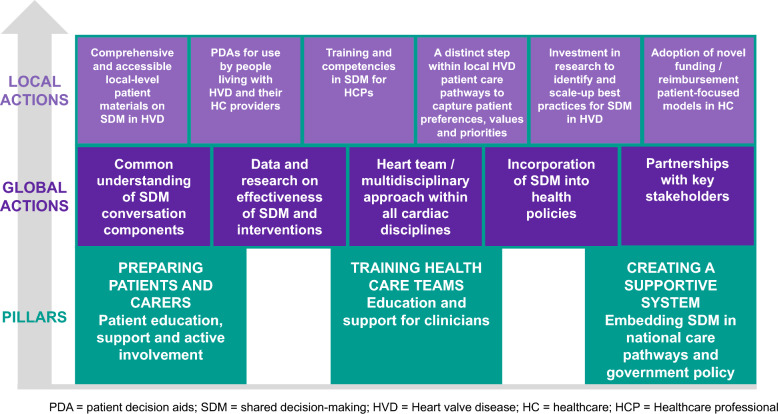
Summary of consensus recommendations of the foundational pillars and global and local actions to accelerate the implementation of shared decision-making in valvular heart disease

We collectively identified opportunities to integrate SDM activities from detection to treatment in the patient pathway (Fig. 2) and mapped the joint recommendations to three guiding pillars: (1) preparing patients and carers, (2) training healthcare teams and (3) creating a supportive system (Fig. 3).

## Discussion

This is the first report of global joint recommendations to support the implementation of SDM in the continuum of care for valvular heart disease co-led and co-developed by people with lived experience, multidisciplinary clinicians and researchers. The proposed calls to action and roadmap highlight key considerations, proposed implementation strategies and preliminary ways to measure impact that will equip patients and clinicians, accelerate systems transformation and sustain the required changes to culture of care, thereby closing the gap between guideline recommendations, clinical care and patient empowerment in the rapidly changing field of valvular heart disease.

In a recent review of SDM in multidisciplinary team-based cardiovascular care, clinicians and researchers provided important insights to determine factors influencing adoption and implementation at the interpersonal, organizational and environmental levels and highlighted the continuum of adoption among multiple moving parts, including patients, clinicians, team working, clinical pathways, policy and research [7]. This concept aligns with our recommendations and further validates the importance of the proposed pillars underpinning the recommendations to sustain the uptake of SDM in valvular heart disease. We examine these priorities in turn.

### Preparing patients and caregivers

Our project placed the highest priority on patients’ perspectives and insights to inform the proposed recommendations. The use of SDM presents a shift in culture, supporting deliberate and active involvement of patients as experts in their personal circumstances, how their health condition impacts their daily life and their goals of care, whilst also refuting historical and often patronizing and paternalistic conventions that allow passive acceptance of physicians’ recommendations based on evidence alone [34]. Importantly, this shift must also consider the principles of diversity, cultural sensitivity and humility and refine and tailor health communication to align with expectations of diverse cultural backgrounds [35, 36].

To this end, patients and their carers ought to be supported to follow a SDM journey that considers their culture of care but may need to recalibrate their beliefs about the role they and their healthcare team play. The pressing need to improve the availability of high-quality, evidence-based decision aids across international regions recommended by our policy panel is of pivotal importance to achieve this goal [37]. These patient-facing visual tools explicitly state the decision to be made, provide information about options (that include the option of maintaining the status quo) and the outcomes associated with each option and help patients clarify their values and preferences [13, 38]. These tools can be used ahead of, or within the clinical consultation, and guide patients’ deliberation alone or in collaboration with others [16]. Education is required to remind clinicians that the use of patient decision aids does not significantly lengthen consultation times. Patients exposed to these tools are more informed about their condition and treatment with realistic expectations and are more active in decision-making processes [16].

This enhanced exposure to resources and information must be augmented by tailored teaching and so-called information coaching. For example, the impact of “Patient Navigators”  for patients undergoing cancer care has been shown to increase the effectiveness of patient decision aids and strengthen SDM in oncology programs [39]. Similarly to the setting of cancer care, the journey of care for people living with valvular heart disease includes multiple time points and decision crossroads that require information and navigation. The role of the clinical valve coordinator (e.g. valve clinic coordinator, valve programme clinician, structural heart disease nurse, transcatheter aortic valve implantation [TAVI] coordinator) can play an essential role in supporting patients and their carers in preparing for SDM – alongside other expert patient educators, clinicians with competencies in communication and person-centred processes, and members of the multidisciplinary heart team [34]. The clinical valve coordinator may be ideally positioned and trained to tailor education and communication that will optimize patients’ preparation for SDM.

### Training healthcare teams

The conduct of SDM requires unique clinical competencies – including best practices in communication and engagement with patients, provision of equity-oriented and culturally competent care, and attitudinal skills to engage in the provision of information and listening to care goals [7, 41]. These efforts require cognitive, emotional and relational competencies and an underpinning goal of ensuring patients’ autonomy [42].

Efforts are needed to advocate and partner with academic institutions to provide guidance and teaching modules that can socialize SDM into curricula and weave competencies into training programmes across all health disciplines [43]. In a 2018 scoping review of the implementation of SDM in undergraduate medical programmes, studies suggested that students’ skills and confidence in having SDM conversations (and their attitudes towards inviting patients to engage in these interactions) improved following training [44]. In contrast, the quality of teaching and education resources to support students remains unsatisfactory [45]. Even after receiving formal training and despite positive intentions, many clinicians report a failure to integrate SDM in their practice for various reasons. These include environmental barriers related to workload, feeling overwhelmed, other priorities, patient caseloads and clinic workflow inefficiencies, and limitations in training and self-perceived competence [46]. Similarly, although established physicians express positive attitudes towards SDM [47], tailored strategies are needed to equip senior clinicians with improved training and guidance and dispel the misperception that an excessive burden of time is required to engage in this process [48].

### Creating a supportive system

The integration of SDM into clinical processes remains one of the main implementation challenges. Research that considers patient, clinician and health service barriers and facilitates the standardized and sustained use of SDM is lacking, especially across regions and within complex health systems. The use of policy incentives remains in its infancy. In 2007, the state of Washington (United States) passed legislation to promote SDM as an alternative to informed consent for preference-based elective treatment decisions (e.g. hip or knee replacement for osteoarthritis) and certified patient decision aids for select conditions. Subsequently, the United States Centers of Medicare and Medicaid Services decided that SDM would be a pre-condition to payment for interventions in stable ischaemic heart disease and valvular heart disease, as well as four other high-volume procedures [49]. These measures illustrate how policy levers may be used to drive changes in health service delivery [50]. The availability of tailored system enablers – including care delivery models, health workforce, better data and research, and funding models – may offer effective strategies to strengthen the implementation of SDM in complex systems.

### Implementation science needed

The GHH roadmap to the implementation of SDM in valvular heart disease provides new guidance co-constructed with patients and reflects global and local priorities, challenges and opportunities. This foundational knowledge can now inform future research to identify optimal strategies for adoption and impact. Implementation science refers to the study of deliberately selected, theory-guided methods to promote the systematic uptake of research findings in clinical, organizational and/or policy contexts [51], with a focus on how and why an intervention was effective (or not) [52]. Our collective intent is to ensure that this knowledge moves “off the shelves, and onto the streets” to move the SDM agenda forward to improve the care of people with valvular heart disease [27].

In our discussions, we gained a growing appreciation that unique issues in low- and middle-income countries were not adequately addressed in the scope of our project. Factors such as late presentation in the progression of valvular heart disease related to systemic issues (i.e. absence of adequate patient journey, timely access to care, awareness and diagnosis), and the impact of cultures of care (i.e. cultural barriers to clinicians’ readiness for shared decision-making, approaches to patient–physician relationships) are significant and unresolved issues that are essential to the development of a truly global approach. This constitutes a call to action for future initiatives.

## Conclusions

Construction of a consensus roadmap for SDM under the co-leadership of clinicians and people with lived experience, combined with the active engagement of patients, researchers and advocacy organizations, has produced a novel and timely report that is intentionally tailored to accelerate global uptake and support local initiatives. The project was limited by the suboptimal representation of participants from low- and middle-income countries. Future initiatives will be necessary to address this significant gap. Accelerated knowledge translation and further research targeting this field are destined to improve the experiences and outcomes of people with valvular heart disease.

## Notes

Generative artificial intelligence was not used in any aspects of this work.

## Data Availability

No datasets were generated or analysed during the current study.
